# Promoting LGBT health and wellbeing through inclusive policy development

**DOI:** 10.1186/1475-9276-8-18

**Published:** 2009-05-15

**Authors:** Nick J Mulé, Lori E Ross, Barry Deeprose, Beth E Jackson, Andrea Daley, Anna Travers, Dick Moore

**Affiliations:** 1 School of Social Work, York University, Toronto, Canada; 2 Social Equity & Health Research Section, Centre for Addiction and Mental Health (CAMH), Department of Psychiatry, University of Toronto, Toronto, Canada; 3 Ottawa LHIN Representative, Rainbow Health Ontario, Ottawa, Canada; 4 Coordinator, Social Determinants of Health Stream, Ontario Rainbow Health Partnership Project, Toronto, Canada; 5 Rainbow Health Ontario, Sherbourne Health Centre, Toronto, Canada; 6 Seniors Program, 519 Church Street Community Centre, Toronto, Canada

## Abstract

In this paper we argue the importance of including gender and sexually diverse populations in policy development towards a more inclusive form of health promotion. We emphasize the need to address the broad health and wellbeing issues and needs of LGBT people, rather than exclusively using an illness-based focus such as HIV/AIDS. We critically examine the limitations of population health, the social determinants of health (SDOH), and public health goals, in light of the lack of recognition of gender and sexually diverse individuals and communities. By first acknowledging the unique health and social care needs of LGBT people, then employing anti-oppressive, critical and intersectional analyses we offer recommendations for how to make population health perspectives, public health goals, and the design of public health promotion policy more inclusive of gender and sexual diversity. In health promotion research and practice, representation matters. It matters which populations are being targeted for health promotion interventions and for what purposes, and it matters which populations are being overlooked. In Canada, current health promotion policy is informed by population health and social determinants of health (SDOH) perspectives, as demonstrated by Public Health Goals for Canada. With Canada's multicultural makeup comes the challenge of ensuring that diverse populations are equitably and effectively recognized in public health and health promotion policy.

## Introduction

In this discursive paper we examine the extent of recognition of gender and sexually diverse populations (LGBTs) (The phrase 'gender and sexually diverse populations' and the acronym 'LGBT' both describe the lesbian, gay, bisexual, transsexual, transgender, two-spirit, intersex, queer and questioning individuals and communities who are marginalized because of their gender identity and/or sexual orientation; herein the term 'gender and sexually diverse populations' is used to address the collective identity of these populations, while 'LGBTs' is used to address the individuals that make up these communities) in Canadian public health promotion policy. We review LGBT health and wellbeing issues, examine limitations of existing models such as population health, the SDOH, and public health goals, in light of the lack of recognition of gender and sexually diverse individuals and communities, and provide a critical analysis of the implications of health disparities. We argue that the gender and sexually diverse populations must be explicitly included in policy development for a more inclusive form of health promotion. In contrast to an illness-based focus such as HIV/AIDS, we emphasize the broad and unique health and wellbeing needs of LGBT people employing anti-oppressive, critical and intersectional analyses. Although literature has been drawn from the most part from Canadian sources to address the realities of LBGT Canadians, we also include literature from Australia, the UK and USA, societies in which the socio-political-cultural experiences of gender and sexually diverse populations are similar albeit not identical. We then conclude with recommendations for a more inclusive approach to recognizing gender and sexually diverse populations in Canadian public health promotion models.

### Public Health Policy in Canada

Historically, Canada has been at the forefront in public health. This was demonstrated by the contributions of the Lalonde Report [1], which introduced the concept of 'health promotion', and the Epp Report [2] which further expanded Lalonde's concepts and developed the 'population health' approach. In the Lalonde Report [1], health determinants comprised four 'health fields' which included biology, lifestyles, environments and health care. However, the report was ultimately understood to champion 'lifestyle' interventions focusing on individual rational action and responsibility, while downplaying the impact of social structures on health [3]. Although the Epp Report [2] and the WHO Ottawa Charter for Health Promotion [4] shifted the focus toward 'healthy public policy', which acknowledged structural determinants of health, community health promotion strategies since that time have continued to target individual responsibility for health behaviours (witness HIV/AIDS prevention programs). Thus, a micro level (individualized) lifestyle approach continues to dominate and define Canadian health promotion [5] by targeting 'high risk' populations through large scale campaigns in which interventions promote risk reduction through behaviour change [6]. Therefore, illness and behaviour remain central while sexuality and gender identity as social locations in the broader social health structures simply do not register.

Populations marginalized by gender identity and sexual orientation have, for the most part, been excluded from mainstream health promotion research, policy and practice. Documents submitted to the federal government have attempted to design models that capture targeted populations (e.g. age, sex, socio-economic status, Aboriginals), but have not included sexual minorities [7,8]. Two latter reports included lesbians and bisexual women [9] and gay men [10,11]. Yet in the Canadian blueprint report *Building on Values: The Future of Health Care in Canada *[12] LGBT populations are completely neglected in the discourse on diversity where other minority groups are addressed (including aging Canadians, those dwelling in rural communities, those of lower socio-economic status, ethnic groups, culturally diverse groups, men and women, visible minorities, people with disabilities, and new Canadians).

The Canadian Institute for Advanced Research (CIAR) and members of its Population Health Program were highly influential in the emergence of the population health approach in Canada. This perspective considers "processes by which system-level variables influence the health of populations" [13]. Health Canada went on to describe the approach as a means "to maintain and improve the health of the entire population and to reduce inequalities in health between population groups" [14]. Despite this, there continues to be a heavy emphasis on targeting the 'entire population' with regard to maintaining and improving health and less so on reducing "inequalities in health between population groups" [14]. Critics charge that the population health approach has been narrowly focused on individualized characteristics and processes as measured by large-scale surveys (i.e. National Population Health Survey, Canadian Community Health Survey). This approach has tended to position 'risk factors' only as correlates to individualized attributes and behaviours, consequently holding to account affected/diseased individuals [13,15]. Meanwhile qualitative, community-based research methods and findings are paid very little attention. Because population health as an approach is not addressing social/structural determinants, it provides weak direction on solutions and social change. Population health policies are devoid of guiding values that call for participation, community development or rectifying social injustices [13]. The implications of this are detrimental to those seeking the implementation of health equities and policy change (i.e. the gender and sexually diverse among other populations) [3,13].

### Sexual and Gender Diversity Issues in Canadian Public Health Policy

Simultaneous to the development of the public health models, the Canadian LGBT communities amassed into a social movement. Human rights protections based on sexual orientation were fought for, and won, at the national, provincial and territorial legislative levels across Canada. For example, activists have achieved the use of inclusive language such as 'same-sex partner', accommodations for same-sex relationships, almost all the rights and responsibilities of opposite-sex common-law relationships and the legalizing of same-sex marriage [16,17]. Although the resulting human rights protections are theoretically inclusive of 'services' such as health care, LGBTs for the most part, have not been recognized as an identifiable population within the health care sector [18]. Further, gender identity is absent from most human rights legislation across Canada with the exception of the Northwest Territories and the City of Toronto [19].

A further element in the exclusion of LGBT issues from Canadian public health policy is research methodology. The health and wellbeing issues of gender and sexually diverse Canadians, have for the most part, been identified and taken up from a community-based perspective – generated by participatory action research initiatives in which the LGBT communities were directly involved in the design, methodology, analysis and iteration of the findings. In other words, gender and sexually diverse communities defined for themselves their health and wellbeing issues, needs and concerns [20]. In contrast, Health Canada's epidemiological approach relies heavily on aggregated individual-level survey data, resulting in a standardization of individualized attributes and experiences in the health care system. This fails to consider and analyze social relations and social forces' impact on health [13,15]. Measurement of individual-level data only, ultimately leads to a focus on individual-level interventions, overlooking the structural sources of health inequities.

It is not surprising, then, that LGBT health needs are not captured within this epidemiological, individualized and illness-based approach. An outcome of this limited approach is a disease-based focus (e.g. HIV/AIDS), that primarily focuses on treating rather than addressing vulnerabilities leading to unsafe sexual practices targeting certain epidemiological populations – for example men who have sex with men (MSM), erasing the social context of identities, and rendering invisible self-affirming gay and bisexual men in the process [21-24] (not to mention lesbians, bisexual women, the trans and intersex populations).

The absence of Canadian gender and sexually diverse populations from the population health approach and extended models (SDOH) and goals (e.g. Canada's Public Health Goals 2005) demonstrates the insidiousness of heterosexism (a belief that heterosexuality is the norm and/or superior to all other forms of sexuality, whereas other sexualities may be considered abnormal, unnatural or not considered at all) and cis genderism (cis gender refers to a traditional binary perspective on gender that assigns strict gender roles to males and females without acknowledgment of overlapping gender characteristics, transitioning between genders or not identifying with either of the traditional genders). Such absence operates within the discipline and practice of social policy through the ideology of heterosexuality, in which normative existence and reproduction is based upon traditional heterosexual ideals [25-27].

This ideology of heterosexuality is manifest in social policy as an institutionalized system that normalizes and naturalizes heterosexuality, and that informs structures of everyday life through, for example, marriage, reproduction and parenting. The ideology of heterosexuality is promoted by non-specificity, or rather a 'not naming' within social policy that appears to include all, but that in fact, excludes the lives of gender and sexually diverse [28,29] people. In Canada, the ideology of heterosexuality and the phenomenon of non-specificity have been challenged by LGBT activists, who have argued against an assumed heterosexual citizen underlying public policy. However, with the exception of demands for the recognition of MSM and associated health needs related to HIV/AIDS the institution of heterosexuality continues to be systematically reinforced and perpetuated in Canada's health policy development and implementation (i.e. health promotion programs) that overlook LGBT populations. Public policy developed in this climate of homo-negativity [30-32] implicitly normalizes and naturalizes heterosexuality [33], resulting in a circular process and continued invisibility of the needs of gender and sexually diverse people.

### Health Effects of Discrimination on Gender Identity and Sexual Orientation

The strength and vibrancy of today's gender and sexually diverse communities is a testament to the movement's long and hard-fought battles for inclusion, recognition, equality, equity and ultimately, acceptance in Canadian society [34,35]. The continuing growth and development of the movement reflects the resilience required to withstand the prejudice, discrimination, and stigma that are still inflicted upon these communities. Both individually and systemically, the health effects of discrimination compromise the wellbeing of the gender and sexually diverse populations [36].

It is beyond the scope of this paper to provide a comprehensive review of the health, health care and wellbeing issues of gender and sexually diverse individuals and communities. Jackson et al. [3] have drawn from available review articles and selected non-governmental organization and government project reports to summarize key findings relevant to gender and sexually diverse populations in Canada. Taken together, these sources indicate that there are patterns of health and illness specific to LGBT people that are independent or may be a result of the marginalization and discrimination they experience. These include health issues that are more common among gay men (e.g. certain cancers, alcohol and tobacco use, sexually transmitted infections) [20,37], lesbians (e.g. cervical and ovarian cancers, alcohol and tobacco use, reproductive health issues) [20,37], bisexuals(e.g. STD/I concerns, and particular barriers to accessing health care due to lack of knowledge on the part of health providers) [20,37,38] transgender, transsexual (e.g. lack of access to hormone therapies and publicly funded surgery for gender transition, certain cancers related to hormone replacement therapies, complications from steroid use, complications from surgical interventions, refusal of care for routine health issues) [37,39], and intersex people (lack of education and training of health professionals, non-consensual sex assignment, cis gender pressures, stigma, and withholding of information) [40].

The Ministerial Advisory Committee on Gay and Lesbian Health [41] for the State of Victoria, Australia reports on the health effects for LGBT populations of heterosexism, sexism, and transphobia. The outcomes of these social patterns of discrimination include: violence and persistent threats of violence, discrimination, social marginalization, social invisibility, isolation, self-denial, guilt, and internalized homo/bi/transphobia. These patterns have been noted to produce negative health effects such as increased rates of alcohol and drug use, greater risks for sexually transmitted infections, and high rates of depression and suicide [3]. LGBT peoples' abilities to form and sustain supportive relationships can be negatively impacted by persistent discrimination in the realms of friends and social networks, finding supportive spiritual/faith communities, as well as intimate relationships and parenting [41].

Gender and sexually diverse populations experience reduced access to quality health care and under-utilization of health care services as a result of fear or lack of confidence, due to widespread and persistent individual and systemic discrimination against them [10,20,37,41-45]. Jackson et al. [3] note that negative/prejudiced attitudes on the part of health care providers combined with systemic discrimination leave gender and sexually diverse patients subject to bias, discrimination, and substandard care. Formats of medical history-taking (such as intake and other medical forms) are frequently exclusive of gender and sexually diverse experiences which may discourage the disclosure of gender identity, sexual orientation, and health-related behaviour or circumstances. Consequently, LGBT people may avoid or delay care (e.g. screening for various health conditions) and/or remain silent about important health concerns. Thus, health problems may be undiagnosed, misdiagnosed, and/or left untreated until they are more severe and less amenable to treatment [43]. Compounding these problems is the limited knowledge on the part of health care providers about the gender and sexually diverse population's health issues [3].

**Figure 1:**
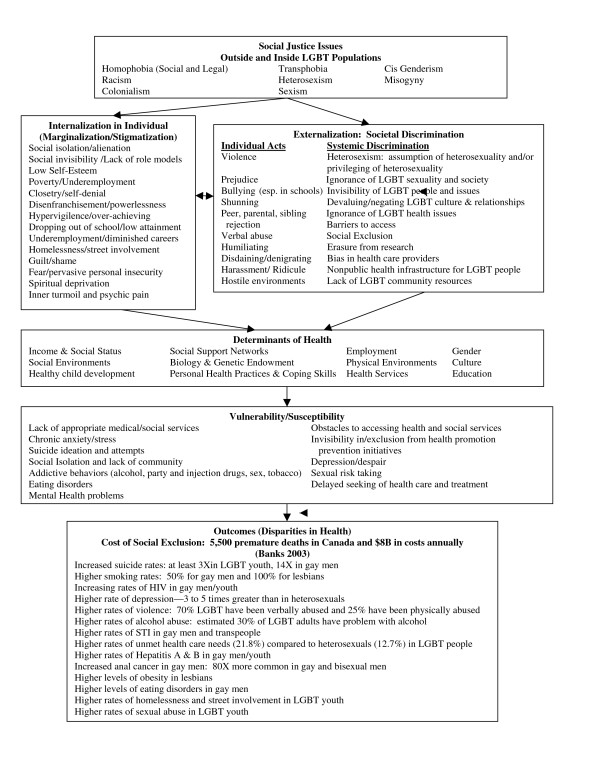
**Structural Framework for Gender and Sexually Diverse Health and Wellbeing Inequities**.

The framework in Figure 1 illustrates, from a structural perspective, the health and wellbeing inequities experienced by gender and sexually diverse populations. From the outset, at the top of the framework, social justice issues are framed within an intersectional discourse acknowledging the multiple social locations and power relationships that LGBT individuals and communities inhabit. These varying social locations intersect with one's gender identity and/or sexuality, and the resulting effects on health and wellbeing. The next two boxes respectively outline internalized and externalized forms of oppression. The former lists affected individualized responses; the latter lists both individually targeted and systemic forms of discrimination with stigmatizing effects. The interaction of individual acts and/or systemic discrimination in the latter has a direct impact on the health and wellbeing of the individual in the former. Further down the framework, specified vulnerabilities and susceptibilities are outlined on individual and systemic levels, indicating repercussions on health and wellbeing for these populations. The box at the bottom of the framework provides the known health disparities for gender and sexually diverse populations. The determinants of health are located at the centre of the framework, midstream between that which ails and the resulting impacts. This framework illustrates the down streaming structural effects that health and wellness inequities have on gender and sexually diverse populations, shifting the focus from individualized pathology to systemic oppression.

### Interactions of the SDOH with Discrimination against Gender and Sexually Diverse Populations

Given the health inequities experienced by gender and sexually diverse populations, it is important to explore how the SDOH recognized in Canadian public health policy affect these populations. Health Canada and the Public Health Agency of Canada name 12 social determinants that impact on health (see middle box labeled 'Social Determinants of Health' in Figure 1), all of which interact with gender identity and sexual orientation. The following examples demonstrate these intersections:

*Income and social status *are key determinants of health for many LGBT people, as their educational achievement and career opportunities can be affected by the prejudice and phobic reactions they experience at school, in the workplace, or elsewhere [44,45]. Studies in the US have refuted the market-created myth regarding 'gay affluence' [46,47]. The economic situation for Canadian gay men and lesbians is complex with the former having personal income 12 percent lower and the latter 15 percent higher than their similarly situated heterosexual counterparts (though both lesbians and heterosexual women, on average, earn less than either heterosexual or gay men). Bisexual men and women earn 30 and 15 percent less than heterosexual individuals, respectively, suggesting that bisexual identity in particular may be associated with socioeconomic deprivation [48]. Poverty is particularly common among people living with HIV/AIDS due to restrictive income-support programs and costly medical therapies [49]. Similarly, trans populations experience extreme social and economic marginalization, not to mention costs of hormone and gender reassignment interventions [37,41,44,45]. Finally, gender and sexually diverse youth who flee or are expelled from abusive homes and who are fearing and/or experiencing rejection have high rates of homelessness [44,45,50]. Moreover, a disproportionate number of street involved youth identify as sexual minorities [51,52].

*Gender *as a social determinant of health has different effects on girls and boys, women and men [53,54]. Much of this is the result of sexism, such that prejudice and phobias directed at gender and sexually diverse people contributes to maintaining a binary system of gender and sexist social relations [45]. Lesbians and bisexual women are doubly affected by inequality [38,41] on the basis of gender and sexual orientation, and 'gender identity' has not been given adequate, if any, consideration as a determinant of health in Canadian health policy, further marginalizing transsexual, transgender, and intersex people.

*'Culture' *as a health determinant is intended to capture experiences of racism and colonialism, yet little research exists on how these intersect with marginalization due to gender identity and/or sexual orientation. Racial and ethnic minority individuals who are also LGBT experience compounded challenges in accessing and utilizing health services [43,45]. Two-Spirit people and First Nations, Inuit and Métis people who identify as gender and/or sexually diverse have been reported to face serious inner conflicts with identity, acceptance, and access to health services [43,55].

*Physical and social environments *can determine the health and wellbeing of LGBT people based upon whether they dwell in rural and remote areas or suburban or major urban centres. Those living in rural and remote communities are reported to experience higher rates of heterosexism and related phobias [43] and fewer adequate services [56]. However, some research suggests that rural gender and sexually diverse communities may compensate by having stronger relations with family and friends, a higher standard of living and less stress compared to gender and sexually diverse people living in urban centres [57].

The *biology and genetic endowment *of gender and sexually diverse people living with a (dis)ability interacts with the prejudice and discrimination they face. They may be presumed to be asexual, may fear coming out to care providers, and may face access barriers in participating in the gender and sexually diverse communities and finding appropriate sensitive health care services for their needs [43].

A lack of adequate *social support networks *negatively affects LGBT people generally and more particularly based on age. Youth struggle with limited funds, fear of rejection, concerns about confidentiality and isolation [37,43]. On the other hand, seniors encounter ageism both within and outside gender and sexually diverse communities, are made to feel invisible and desexualized, fear prejudice and phobias in institutional care, and may have lived through a history in which gender and sexual diversity was pathologized [43,58]. Indeed, many of the first generation of liberated gay men and lesbians feel coerced to return to the closet in their declining years.

Increasingly, members of the gender and sexually diverse communities are questioning the SDOH and the extent to which LGBT concerns are recognized therein [3]. Firstly, these populations are not explicitly represented as an identity group. The social determinants of culture and gender, which were added to Health Canada materials in the late 1990s, could theoretically capture the gender and sexually diverse populations, but a diversified analysis has not been adequately taken up in either area. Although increased health risks for some groups "determined by dominant cultural values that contribute to the perpetuation of conditions such as marginalization, stigmatization ..." [59] have been described as determinants of health related to culture, Aboriginal and immigrant populations are featured, whereas gender and sexually diverse populations are neither mentioned within these featured populations nor discussed independently. Gender focuses on social determinants that affect the health of those that fit traditional binary notions of socially constructed females and males. The effects of gender on health have not included experiences of transsexual, transgender and intersex people. Therefore, despite the health effects of prejudice and discrimination on LGBT populations and the impact of intersections with and among other determinants of health, these populations are ignored in conventional population health models. As a result, they are overlooked when developing health policy – we explore this concern in the next section on Canada's Public Health Goals.

### Applying Canada's Public Health Goals to Gender and Sexually Diverse Populations

In October, 2005, the Public Health Agency of Canada announced a new set of Public Health Goals for Canada. Its intent was to provide a series of guideposts for health improvement and to enhance Canadians' quality of life, and as such was not intended to provide a detailed means of achieving this. The goals consist of the overarching goal that "As a nation, we aspire to a Canada in which every person is as healthy as they can be – physically, mentally, emotionally, and spiritually" and nine additional goals that are sub-sectioned under four categories [60]. Below, we provide examples for ensuring that the nine Public Health Goals for Canada address the concerns of gender and sexually diverse people:

• Addressing the goal of enabling our children and youth, particularly those that identify as gender and/or sexually diverse or are members of same-sex families, to "reach their full potential, growing up happy, healthy, confident and secure" [60] could be supported by initiatives to formalize referral networks, support services, mentoring programs, and inclusive education on diversity [38,39,43,61]. This would require the systemic participation of the health care, social service and education sectors that work with children and youth.

• The goal of ensuring that our social and physical environments are safe and healthy could be supported by establishing government-sponsored, public health campaigns against heterosexism, sexism, cis genderism and various phobias, within and across the health care, social service, housing, and employment sectors [43]. This would merely uphold human rights legislation at both federal and provincial/territorial levels in Canada that at minimum recognizes sexual orientation, if not gender identity.

• Regarding the goal of ensuring that "every person has dignity, a sense of belonging, and contributes to supportive families, friendships and diverse communities" [60] supportive services (e.g. 'help phone lines', community outreach/development initiatives, positive websites and other electronic methods of networking, trans-positive shelters, and transitional housing), could be provided for gender and sexually diverse individuals and their families, while ensuring that existing barriers to services and benefits are removed [38,39,43,61]. This would require the recognition of the gender and sexually diverse in the development and implementation of both policies and services.

• "Life-long learning" could be achieved through continuing education of the general public and of health care and social service providers regarding gender and sexually diverse issues, together with integrated and targeted programs for LGBT individuals and communities (e.g. employment resources such as skills training for trans people) [38,39,43,61]. For a comprehensive approach, this would need to be undertaken both formally and informally at all levels of the education system and in all public health promotion.

• The goal that we all "participate in and influence the decisions that affect our personal and collective health and well-being" [60] highlights the importance of community involvement in not only one's own health and health care, but that of others. Like other population groups, it is imperative that gender and sexually diverse people be directly involved in the planning, design, development, and implementation of research, programs and services, and health care and social service delivery [39,62]. Direct investment is necessary to achieve this goal.

• The goal of making the world "a healthy place for all people, through leadership, collaboration and knowledge" [60] requires improved knowledge production and exchange in the forms of research and information dissemination regarding gender and sexually diverse people's health and well-being. Capacity building strategies should encourage collaborative partnerships between members of the gender and sexually diverse communities, activists, frontline service providers, and other professionals, policy makers and academics to work together towards creating effective educational and prevention-focused health promotion initiatives [38,39,43,61,62].

• Ensuring the goal that every person gets "the support and information they need to make healthy choices" [60] requires gender and sexually diverse-specific health promotion programs. These should provide education and information resources about healthy sexuality and gender variations, and other health issues of relevance to these populations [38,39,43,61]. Additionally, health promotion strategies that are aimed at the general public addressing prejudice, heterosexism and various phobias towards the gender and sexually diverse will go a long way in reducing health disparities.

• The commitment to "prevent and respond to health and safety threats via Canadian and globally coordinated efforts", represents one of the greatest gaps for the gender and sexually diverse populations. Within Canada, a systemic connection needs to be made between human rights protections for the gender and sexually diverse populations and corresponding health and social services [43]. Canada would then be in a better position to promote the inclusion of gender and sexually diverse individuals and communities at the global level.

• The last goal calling for a strong health system that "responds to disparities in health status and offers timely, appropriate care" [60] requires initiatives that create sensitive, equitable and accessible services for gender and sexually diverse people. This would include but not be limited to a systemic recognition of discrimination based on gender identity and sexual orientation as important determinants of health, intake forms that are inclusive and representative, services that are sensitive to the unique stressors that LGBT people experience (e.g. increased attention to the existence of LGBT couples and families, improved access to hormone therapy, psychosocial support and sex/gender reassignment surgeries), and improved anti-oppressive curricula at post-secondary institutions training future health and social service professionals [38,39,43,61,62]. Ideally, an interconnected dialogue needs to be developed and maintained between stakeholders at all levels in order to continuously respond to the ever-changing health needs of these populations.

These examples provide a valuable beginning for understanding, conceptualizing and developing a discourse of public health that captures objectives, indicators, targets and strategies that are inclusive of LGBT individuals and communities. The links between the health and wellbeing issues of gender and sexually diverse populations and Canada's new Public Health Goals offer a blueprint of the kind of initiatives and activities that must take place in order to ensure the overarching goal, that "every person is as healthy as they can be – physically, mentally, emotionally, and spiritually" [60].

## Discussion: towards an inclusive approach

In this paper, we have illustrated how Canada's new Health Goals can connect to the health and wellbeing issues affecting gender and sexually diverse people. Such connections now need to be acknowledged and applied in the development and implementation of public health policy. To date, Canada (along with parts of the UK and the USA) has failed to capture gender and sexually diverse populations in health and social service public policies [18], despite evidence for numerous structurally-driven, population-based health disparities [3]. LGBT people are generally not captured in the Canadian lexicon of 'visible minorities' [23]. This exclusion is at great cost: lack of recognition of gender and sexually diverse populations by the health care system has been estimated to result in close to 5,500 premature deaths in Canada and $8B in annual costs [42].

LGBT people cross all socio-economic, ethno-racial, age, gender, (dis)ability, religious, geographical location, educational, and relationship status lines. Consequently, for many in these communities, their existence is made up of multiple intersecting social identities. These identities intersect and are affected by societal power dynamics that can result in oppressions and/or privileges that play out structurally (macro) or individually and interpersonally (micro) [63]. The anti-oppression approach arose from social justice movements (inclusive of the queer movement) that have challenged hegemonic social structures and norms and emphasized the intersectional realities of diverse populations [3,64] with a goal of achieving social justice. Institutions with power at the macro level will often use such social identities to oppress leaving individuals at the micro level, few if any supports [65,66] (for example, in services for women that do not take into consideration race, class, sexual orientation, or gender identity, among others). Recognition of these dilemmas and how they affect LGBT populations and their social identities and health and wellbeing requires us to reframe population health, the SDOH, and public health goals to provide a stronger foundation for inclusive health promotion policies and initiatives that capture gender and sexual diversity.

**Figure 2:**
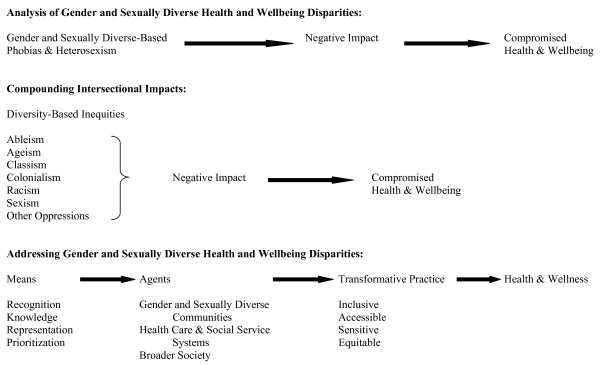
**Gender and Sexual Diversity Health and Wellbeing Critical Analysis**.

Figure 2 illustrates a critical analysis of gender and sexual diversity health and wellbeing in three phases, as a means of expanding current discourse on public health promotion to be inclusive of these populations, and applying such discourse to the development of LGBT-positive health promotion policy. Each phase is displayed on a progressive continuum indicating impact or process required to address this population's health and wellbeing issues. In the first phase, an analysis is provided that outlines the negative impact of phobias and heterosexism on the health and wellbeing of LGBT people with compromised health and wellbeing outcomes. In other words, experiencing homo/bi/transphobia, heterosexism, and/or cis genderism negatively affects the health and wellbeing of these communities' members. The second phase acknowledges the diversity of LGBT populations and how multiple oppressions compound negative health effects and compromise health and wellbeing outcomes. Although the focus of this paper necessitates the placing of an analysis of phobias, heterosexism and cis genderism in the first phase, diverse members of LGBT communities may perceive/experience/prioritize the effects of oppression based on multiple identities differently.

The third and final phase of figure 2 provides a progressive continuum by which health and wellbeing disparities experienced by gender and sexually diverse populations can be addressed. This continuum begins with interventions to challenge and expand public health promotion discourse to reflect these populations. There must be recognition of the unique and specific health and wellbeing issues affecting LGBT people, substantiated with knowledge guided by a critical/structural analysis as outlined in the previous two phases. Beyond informing discourse, the next two elements of the framework address implementation, in which LGBT individuals and communities be represented in policy, funding, programming, and services, prioritizing them in order to address their health and wellbeing concerns. For this to succeed three agents must mobilize these issues: first and foremost, the gender and sexually diverse communities' voices and experiences must define the issues and set the context; second, health care and social service systems must reshape how they provide service delivery; and third, it is important that broader society become knowledgeable and sensitized to these issues via public health education campaigns in order to combat phobias, heterosexism and cis genderism directed at these populations. Associated agents such as policy makers, funders and postsecondary educational institutions, and professional associations that train health care and social service professionals would also need to contribute to this process. An engagement and commitment to the process of addressing these concerns on the part of these agents would contribute to a transformative practice in which service provision would become more inclusive, accessible, sensitive, and equitable towards the gender and sexually diverse with a positive health and wellness outcome.

## Conclusion

In this paper we have described a series of perspectives, ideas, and concepts to expand public health promotion discourse in order to be inclusive of the gender and sexually diverse populations. We have highlighted a series of health and wellbeing inequities and disparities unique to these populations, providing a structural framework that illustrates the downstream effects. The SDOH, population health perspective and the public health goals of Canada were critically examined as current models of public health promotion, revealing shortcomings and limitations that in effect exclude LGBT people and communities. This is not to say that the current models cannot be modified to be inclusive of the gender and sexually diverse, as was illustrated via the expanded public health goals posited. By employing anti-oppressive, critical, and intersectional analyses to examine, deconstruct and challenge these models, a more expansive and inclusive discourse emerges that makes room for and can accommodate populations currently excluded. We have also offered an upstream analysis that proposes a method of understanding health and wellbeing disparities experienced by LGBT populations, the compound health effects of intersecting oppressions and a systemic means of addressing them. Thus, we argue that an expansion of public health promotion discourse that would recognize gender and sexually diverse populations, together with a reframing of the SDOH, population health, and Canada's public health goals, would contribute to the development and implementation of more inclusive, diversified public health promotion policies that will benefit LGBT populations and communities – as well as Canada' public health system.

## Competing interests

The authors declare that they have no competing interests.

## Authors' contributions

NJM, BEJ, LER, AT, AD and DM engaged in a collective process as part of the SDOH stream of the Ontario Rainbow Health Partnership Project (ORHPP), all contributing to the literature review and content analysis. BEJ and NJM prepared and led the consultative forum held at a read Canadian Rainbow Health Coalition (CRHC) national conference. NJM and BD developed the ‘Structural Framework for Gender and Sexually Diverse Health and Wellbeing Inequities’ and the ‘Gender and Sexual Diversity Health and Wellbeing Critical Analysis,’ with input from the CRHC board and all members of the SDOH stream of ORHPP. NJM drafted the manuscript with input from LER, BD, BEJ, AD and AT. NJM contributed to and coordinated the revisions received from LER, BD, AD, BEJ and AT. NJM and AD provided interim formatting with final revisions by NJM, LER and BD. This work draws on a previously released report entitled "Whose Public Health? An Intersectional Approach to Sexual Orientation, Gender Identity and the Development of Public Health Goals for Canada" (2006) [3]. BEJ was the lead author on this report; NJM, LER, AT, AD, and DM were members of the Advisory Committee that provided direction for the report and are listed as co-authors. All authors read and approved the final manuscript.

## References

[B1] Lalonde M (1981). A New Perspective on the Health of Canadians.

[B2] Epp J (1986). Achieving Health for All: A Framework for Health Promotion.

[B3] Jackson B, Daley A, Moore D, Mulé N, Ross L, Travers A, Montgomery E (2006). Whose Public Health? An Intersectional Approach to Sexual Orientation, Gender Identity and the Development of Public Health Goals for Canada.

[B4] World Health Organisation, Health and Welfare Canada, Canadian Public Health Association (1986). Ottawa Charter for Health Promotion.

[B5] Raphael D (2000). The question of evidence in health promotion. Health Promotion International.

[B6] Raymond J (1989). Behavioural epidemiology: The science of health promotion. Health Promotion.

[B7] Federal, Provincial, and Territorial Advisory Committee on Population Health (1994). Strategies for Population Health: Investing in the Health of Canadians.

[B8] Federal, Provincial, and Territorial Advisory Committee on Population Health (1996). Report on the Health of Canadians.

[B9] Advisory Committee on Women's Health Surveillance (1999). Women's Health Surveillance: A Plan of Action for Health Canada.

[B10] National Reference Group (2000). Framing Gay Men's Health in a Population Health Discourse: A Discussion Paper.

[B11] National Reference Group (2000). Valuing Gay Men's Lives: Reinvigorating HIV Prevention in the Context of our Health and Wellness.

[B12] Romanow RJ (2002). Building on Values: The Future of Health Care in Canada – Final Report.

[B13] Raphael D, Bryant T (2002). The limitations of population health as a model for a new public health. Health Promotion International.

[B14] Health Canada (1998). Taking action on population health: A position paper for health promotion and programs branch staff.

[B15] Jackson BE (2003). Situating epidemiology. Advances in Gender Research, 7 (Gender perspectives on health and medicine: Key themes).

[B16] Cossman B (2002). Sexing citizenship, privatizing sex. Citizenship Studies.

[B17] Petersen C, Rosenbloom R (1996). Canada. Unspoken Rules: Sexual Orientation and Women's Human Rights.

[B18] Mulé NJ, Badgett L, Frank J (2007). "Sexual Orientation Discrimination in Health Care and Social Service Policy: A Comparative Analysis of Canada, the UK and USA". Sexual Orientation Discrimination: An International Perspective.

[B19] Rainbow Health Network (2008). Trans Rights are Human Rights!: Canadian Human Rights Act Amendment (Gender Identity).

[B20] Ryan B, Brotman S, Rowe B (2000). Access to care: Exploring the health and well-being of gay, lesbian, bisexual and two-spirit people in Canada.

[B21] Adam B (2007). Commentary on lack of focus on gay men at the 2006 World AIDS Conference in Toronto, made at the National Strategic Research Cluster Development Meeting on Gender and Sexual Diversity and Health and Wellbeing, Toronto.

[B22] Deeprose B (2002). Unpublished Why "Gay Men" not "MSM" Ottawa.

[B23] Mulé NJ (2005). "Beyond Words in Health and Wellbeing Policy: 'Sexual Orientation' – From Inclusion to Infusion". Canadian Review of Social Policy.

[B24] Young R, Meyer I (2005). The trouble with 'MSM' and 'WSW': Erasure of the sexual minority person in public health discourse. American Journal of Public Health.

[B25] Carabine J, Wilson AR (1995). Invisible sexualities: sexuality, politics and influencing social policy making. A Simple Matter of Justice.

[B26] Carabine J (1996). A straight playing field or queering the pitch? Centring sexuality in social policy. Feminist Review.

[B27] Carabine J, Taylor D (1996). Constructing women: Women's sexuality and social policy. Critical Social Policy: A Reader.

[B28] Carabine J, Richardson D (1996). Heterosexuality and social policy. Theorizing Heterosexuality: Telling it Straight.

[B29] Carabine J, Jean Carabine (2004). Sexualities, personal lives and social policy. Sexualities: Persona Lives and Social Policy.

[B30] Blumenfeld W, Raymond D (1993). Looking at Gay and Lesbian Life.

[B31] Russel GM, Bohan JS, Russel GM, Bohan JS (1999). Implications for public policy. Conversations about Psychology and Sexual Orientation.

[B32] Carabine J, Lewis G, Gewirtz S, Clarke J (2000). Constituting welfare subjects through poverty and sexuality. Rethinking Social Policy.

[B33] Richardson D (1996). Heterosexulity and social theory. D Richardson Theorizing Heterosexuality.

[B34] Smith M (1999). Lesbian and Gay Rights in Canada: Social Movements and Equality-Seeking, 1971 – 1995.

[B35] Warner T (2002). Never Going Back: A History of Queer Activism in Canada.

[B36] Meyer IH (2001). Why Lesbian, Gay, Bisexual, and Transgender Public Health?. American Journal of Public Health.

[B37] Dean L, Meyer IH, Robinson K, Sell RL, Sember R, Silenzio VMB, Bowen DJ, Bradford J, Rothblum E, Scout, White J, Dunn P, Lawrence A, Wolfe D, Xavier J (2000). Lesbian, gay, bisexual, and transgender health: Findings and concerns. Journal of the Gay and Lesbian Medical Association.

[B38] Dobinson C, MacDonnell J, Hampson E, Clipsham J, Chow K (2003). Improving the Access and Quality of Public Health Services for Bisexuals.

[B39] Gapka S, Raj R (2003). Trans Health Project.

[B40] Tamara A (1997). The medical management of intersexed children: An analogue for childhood sexual abuse.

[B41] Ministerial Advisory Committee on Gay and Lesbian Health (2003). A Health and Wellbeing Action Plan for Gay, Lesbian, Bisexual, Transgender and Intersex Victorians.

[B42] Banks C (2003). The Cost of Homophobia: Literature Review on the Human Impact of Homophobia in Canada.

[B43] Coalition for Lesbian and Gay Rights in Ontario (1997). Systems Failure: A Report on the Experiences of Sexual Minorities in Ontario's Health-Care and Social-Services Systems.

[B44] INCLUSION Project (2003). Towards a Healthier LGBT Scotland.

[B45] Ryan B, Chervin M (2000). Framing Gay Men's Health in a Population Health Discourse.

[B46] Badgett MVL (1998). Income inflation: The myth of affluence among gay, lesbian, and bisexual Americans.

[B47] Gates G (2003). Income of gay men lags behind that of men partnered with women.

[B48] Carpenter CS (2008). Sexual orientation, work, and income in Canada. Canadian Journal of Economics.

[B49] Canadian Association of Social Workers (CASW) (2001). Social Work Manifesto on HIV/AIDS. Canadian Social Work.

[B50] Duncan K, Clipsham J, Hampson E, Krieger C, MacDonnell J, Roedding D, Chow K, Milne D (2000). Improving the Access to and Quality of Public Health Services for Lesbians and Gay Men.

[B51] Gaetz S (2004). Safe streets for whom? Homeless youth, social exclusion, and criminal victimization. Canadian Journal of Criminology and Criminal Justice.

[B52] Gaetz S, O'Grady B, Vaillancourt B (1999). Making money: The Shout Clinic report on homeless youth and employment.

[B53] Armstrong P, Amaratunga C, Bernier J, Grant KG, Pederson A, Willson K, (eds) (2001). Exposing privatization: women and health care reform in Canada.

[B54] Health Canada (1999). Health Canada's Women's Health Strategy Cat H221-138/1997.

[B55] Brotman S, Ryan B (2004). An intersectional approach to queer health policy and practice: Two-Spirit people in Canada. Canadian Diversity.

[B56] Ross LE, Goldfinger C, Julien D, Levy JL (2007). Insémination artificielle, grossesse et expériences parentales de lesbiennes vivant en régions peu habitées. Homosexualités: Réalités régionales.

[B57] Oswald RF, Culton LS (2003). Under the rainbow: Rural gay life and its relevance for family providers. Family Relations: Interdisciplinary Journal of Applied Family Studies.

[B58] Brotman S, Ryan B, Meyer E, Chamberland L, Cormier R, Julien D, Peterkin A, Richard B (2006). The health and social service needs of gay and lesbian seniors and their families in Canada.

[B59] Public Health Agency of Canada (2004). Population health: What determines health?.

[B60] Public Health Agency of Canada (2005). Health Goals for Canada.

[B61] Winnipeg Gay/Lesbian Resource Centre (1996). Breaking Barriers.

[B62] Sum Quod Sum Foundation (1997). A Report on the Needs Assessment Survey of Senior Gays and Lesbians.

[B63] Moosa-Mitha M, Brown L, Strega S (2005). Situating anti-oppressive theories within critical and difference-centred perspectives. Research as Resistance: Critical, Indigenous and Anti-oppressive Approaches.

[B64] Mulé NJ (2006). "Equality's Limitations, Liberation's Challenges: Considerations for Queer Movement Strategizing". Canadian Online Journal of Queer Studies in Education.

[B65] Pharr S (1988). Homophobia: A weapon of sexism.

[B66] Weber L, Parra-Medina D (2003). Intersectionality and women's health: charting a path to eliminating women's health disparities. Advances in Gender Research 7 (Gender Perspectives on Health and Medicine: Key Themes).

